# Genetic and environmental influences on food preferences in adolescence^1^,^2^

**DOI:** 10.3945/ajcn.116.133983

**Published:** 2016-07-06

**Authors:** Andrea D Smith, Alison Fildes, Lucy Cooke, Moritz Herle, Nicholas Shakeshaft, Robert Plomin, Clare Llewellyn

**Affiliations:** 3 Health Behaviour Research Centre, Department of Epidemiology and Public Health, University College London, London, United Kingdom;; 4 Great Ormond Street Hospital, Children’s NHS Foundation Trust, London, United Kingdom; and; 5 Medical Research Council Social, Genetic, and Developmental Psychiatry Centre, Institute of Psychiatry, Psychology, and Neuroscience, King's College London, London, United Kingdom

**Keywords:** food preferences, behavioral genetics, heritability, twin study, cohort study

## Abstract

**Background:** Food preferences vary substantially among adults and children. Twin studies have established that genes and aspects of the shared family environment both play important roles in shaping children’s food preferences. The transition from childhood to adulthood is characterized by large gains in independence, but the relative influences of genes and the environment on food preferences in late adolescence are unknown.

**Objective:** The aim of this study was to quantify the contribution of genetic and environmental influences on food preferences in older adolescents.

**Design:** Participants were 2865 twins aged 18–19 y from the TEDS (Twins Early Development Study), a large population-based cohort of British twins born during 1994–1996. Food preferences were measured by using a self-report questionnaire of 62 individual foods. Food items were categorized into 6 food groups (fruit, vegetables, meat or fish, dairy, starch foods, and snacks) by using factor analysis. Maximum likelihood structural equation modeling established genetic and environmental contributions to variations in preferences for each food group.

**Results:** Genetic factors influenced a significant and substantial proportion of the variation in preference scores of all 6 food groups: vegetables (0.54; 95% CI: 0.47, 0.59), fruit (0.49; 95% CI: 0.43, 0.55), starchy foods (0.32; 95% CI: 0.24, 0.39), meat or fish (0.44; 95% CI: 0.38, 0.51), dairy (0.44; 95% CI: 0.37, 0.50), and snacks (0.43; 95% CI: 0.36, 0.49). Aspects of the environment that are not shared by 2 twins in a family explained all of the remaining variance in food preferences.

**Conclusions:** Food preferences had a moderate genetic basis in late adolescence, in keeping with findings in children. However, by this older age, the influence of the shared family environment had disappeared, and only aspects of the environment unique to each individual twin influenced food preferences. This finding suggests that shared environmental experiences that influence food preferences in childhood may not have effects that persist into adulthood.

## INTRODUCTION

A healthy and balanced diet is central to optimal health in both the short and long term. Food preferences are important drivers of actual food choice, determining micro- and macronutrient intakes (1–3). Poor dietary quality increases the risk of nutrition-related chronic disease, obesity (4, 5), and associated comorbidities such as type 2 diabetes (6). Understanding the etiology of food preferences therefore has important implications for policy makers and clinicians.

Twin studies have established the relative importance of genetic compared with environmental influences on food preferences in adults and children (7). A recent study in 3-y-old British children suggested moderate heritability for liking of vegetables (0.54), fruit (0.53), protein foods (0.48), snacks (0.29), starches (0.32), and dairy foods (0.27) (8). Likewise, an earlier study in 4-y-old British twins (9) found that liking of fruit (0.51), vegetables (0.37), protein foods (0.78), and dessert-type foods (0.20) all had some genetic basis, albeit with varying heritability estimates as expected from the smaller sample size. Importantly, in both studies, it was the effect of the environment shared by 2 twins in a family (the “shared environment” e.g., being raised in the same household) that influenced food preferences, with minimal contribution from environmental influences that are unique to each child (the “nonshared environment”). This makes sense given the importance of the home family environment (e.g., food availability) for the eating behavior of preschool children (10), because the family setting is the primary environment within which a child develops his or her behaviors (11).

Studies in adult twins have also shown that food preferences tend to have a moderate genetic basis; however, the unique environment is the most important influence on adult food intake and choice (12–14), with little evidence of a meaningful influence by the shared environment (12). This indicates that shared environmental factors that play a role in shaping the development of food preferences in childhood are less important in adulthood, but it is unclear at what stage the influence of the shared environment declines. To our knowledge, there are no existing studies of the relative influence of genes and shared and unique environmental factors on the food preferences of older adolescents. This is an important developmental transition into adulthood that is characterized by gains in independence; at the same time, the family remains an important but diminishing source of influence as adulthood approaches.

In this study we investigate the relative magnitude of genetic, shared, and unique environmental influences on food preferences in a large sample of older adolescents (18–19 y of age). We hypothesized that food preferences would have a moderate genetic basis, in keeping with studies in both children and adults, and be influenced by both the shared and unique environment.

## METHODS

### Sample

Study participants were twins from the Twin Early Development Study (TEDS),^6^ a birth cohort of 16,810 families with twins born in England and Wales during 1994–1996. TEDS was previously shown to be reasonably representative of the general population (15). For the current study, twins were from a subsample of twin pairs born between September 1995 and August 1996. Requests to complete the online food preference questionnaires were sent out to the entire subsample (3166 pairs; *n* = 6332 individuals) by letter and e-mail. Subjects were offered a £10 voucher to complete the survey, resulting in 3155 individual twins who consented to participate. Data from twins with serious medical or perinatal problems or with unknown sex or zygosity were excluded (*n* = 290). Of these, 52 (17.9%) were monozygotic, 156 (58.6%) were dizygotic, and 82 (21.8%) were of unknown zygosity. This breakdown is representative of typical monozygotic/dizygotic proportions observed in twin populations. Importantly, health status (a factor that conceivably influences food preferences; χ^2^ = 5.918, *P* = 0.15), food restrictions (χ^2^ = 0.26, *P* = 0.87), or BMI (*t* = 0.45, *P* = 0.65) did not differ by zygosity between the excluded individuals. The final sample consisted of 2865 individuals, representing 1010 complete monozygotic pairs, 909 dizygotic same-sex, and 946 dizygotic opposite-sex pairs. In addition, data were included from 379 unpaired individuals, with 90 from monozygotic, 107 from dizygotic same-sex, and 182 from dizygotic opposite-sex pairs. The procedures followed were in accordance with King’s College London ethical standards on human experimentation, and approval was obtained from the relevant committee on human subjects.

### Measures

#### Sociodemographic measures and zygosity

Date of birth, sex, birth complications, and socioeconomic information were collected in the baseline questionnaire. BMI was calculated from self-reported weight and height squared (kg/m^2^). Zygosity had previously been collected by using a parental report questionnaire completed in early childhood. DNA analysis has shown the questionnaire to be >95% accurate (16); uncertain zygosity was determined from DNA.

#### Food preferences

Food preferences were measured via a self-report questionnaire that asked participants to rate their liking of 69 individual foods on a 5-point Likert scale, ranging from “not at all” to “a lot” a higher score was indicative of greater liking of a food. Participants were instructed to select the “not applicable” option for foods that they had never tried. The food preference questionnaire was based on a previous questionnaire that was used to establish genetic and environmental influences on food preferences of 4-y-old children (17) to allow comparison between estimates derived from this and the previous study. For this study, revisions of the original questionnaire included the elimination of outdated food items (e.g., blancmange), the omission of composite dishes (e.g., pizza), and the addition of more contemporary foods commonly consumed by older adolescents and young adults (e.g., hummus). Principal components analysis in SPSS version 22 produced food-group factors comparable to the original food groupings (8, 9, 18). Because food preference factors are expected to correlate, an oblique rotation method was chosen (Direct oblimin).

A previous test-retest was undertaken in a sample of the twins’ siblings to assess the reliability of the food preference questionnaire over a 2-wk period. Siblings (*n* = 205) were invited to complete the online questionnaire, with *n* = 94 participants completing both waves of data collection. Mean food preference score test-retest coefficients ranged from 0.61 to 0.95, which showed the questionnaire to be reasonably stable. Internal reliability (indexed by using Cronbach’s α) was reasonable for the following food groups: vegetables (spinach, carrots, green beans, etc.; α = 0.89; 18 items), fruit (oranges, grapes, apples, etc.; α = 0.84; 7 items), meat or fish (beef, lamb, chicken, etc.; α = 0.81; 12 items), dairy (hard cheese, cream, yogurt, etc.; α = 0.77; 10 items), snacks (chips, cake, chocolate, etc.; α = 0.80; 9 items), and starch (bread, porridge, rice, etc.; α = 0.68; 6 items).

#### Food restrictions

Twins were asked whether they follow a pescatarian, vegetarian, or vegan dietary regimen. In addition, food allergy information was ascertained by using a self-completed food allergies checklist.

### Statistical analysis

Twin studies are able to provide estimates of genetic influence on traits, but importantly, they are also able to separate out environmental influences into the following: *1*) those that are completely shared between 2 twins in a pair and contribute to twin similarity over and above genetics (e.g., living in the same household) and *2*) influences that are unique to each individual twin (i.e., unshared between 2 twins in a pair) and contribute to differences between twins (e.g., having different friends). Monozygotic twins are 100% genetically correlated, whereas dizygotic twins are ∼50% similar genetically; however, both types of twins share their environments to a similar extent. This means that resemblance between monozygotic and dizygotic twins for an observable trait (e.g., food preferences) can be compared to provide an estimate of genetic influence (indexed by the effect size indicator called “heritability,” which describes the proportion of total variance that can be attributed to inherited DNA differences) and shared and unique environmental effects. Greater similarity between monozygotic twins than between dizygotic twins indicates a genetic contribution to trait variation, because researchers assume that the only difference between monozygotic and dizygotic twins is that monozygotic twins are twice as similar genetically, and their environments are shared equally (19). The extent to which monozygotic twins are different from one another provides a direct estimate of the unique environment, the only source of monozygotic pair difference (because their similarity reflects both shared genes and shared environments) other than error of measurement. Each component of variance ranges from 0% to 100%, indicating the proportion of total variation (individual differences) attributable to variation in each of genetic, shared, and unique environmental influences.

Two approaches were used to quantify the relative influence of genetic, shared, and unique environmental influences on food preference variation. Initially, food item and food category intraclass correlations (ICCs) were calculated for both monozygotic and dizygotic pairs, which indicate the pattern of the relative importance of genetic, shared, and unique environmental influences on variations in food preference scores. Maximum likelihood structural equation modeling (MLSEM) was used to derive more precise estimates of the 3 sources of variation (with 95% CIs), as well as to provide goodness-of-fit statistics. Additive genetic factors are denoted by “A,” shared environmental factors by “C,” and unique environmental influences by “E” (which also includes measurement error). MLSEM estimates A, C, and E based on the expected structure of the variance-covariance matrices for monozygotic and dizygotic twins, on the basis of the key assumptions of the twin design: for example, monozygotic covariation reflects the fact that the twins share all of their genes and all of their shared environments, rMZ = 1A + 1C; on the other hand, dizygotic covariation reflects the fact that the twins share half of their genes but all of their shared environments, rDZ = 0.5A +1C, where rMZ and rDZ indicate ICCs for monozygotic and dizygotic twin pairs, respectively. In an independent model, the presence of nonadditive genetic effects, denoted by “D,” can be investigated. Shared environment factors and nonadditive genetic effects cannot be estimated at the same time; therefore, A, D, and E factors need to be fitted to the data in a separate model.

Initially, food preference scores were residualized for age and sex effects. This is a standard procedure in twin modeling because all twins share their age exactly (and sex for same-sex twins), and these factors can therefore inflate the shared environment effect (20). First, a saturated model was fitted, which applies no constraints to the data and simply estimates means, covariances, and variances for monozygotic and dizygotic twins. Then, a full ACE model was fitted and compared with the saturated model for goodness-of-fit, as indicated by the likelihood ratio test and the Akaike information criterion (AIC). The likelihood ratio test is a procedure used to select the best-fitting model among hierarchical nested models. The likelihood ratio statistic approximately follows a χ^2^ distribution, and any addition of more variables to a model increases the likelihood score. Comparing the likelihood scores of multiple models allows for objective selection of the significantly superior model fit. Calculation of the AIC statistic penalizes for the addition of additional variables and thereby favors the simplest, most parsimonious model for the observed data. Submodels consecutively dropping the A and C variables (E is never dropped from the model because it includes measurement error) were nested within the full ACE model and the best-fitting model selected, as indicated by the lowest absolute value of the AIC and smallest Δχ^2^. The AIC is also used for comparing nonnested models (i.e., in the case of comparing the fit of ACE and ADE models). Generally, the model with the overall lowest AIC indicates the most parsimonious solution, the best model to explain the structure of the observed data. MLSEM was performed in R (21) by using the structural equation modeling software OpenMx, version 2.2.6 (22).

## RESULTS

Participants’ mean age was 19.1 y (SD = 0.3 y; range = 18.6–19.6 y), and there were slightly more females (59.8%) than males in our sample. One-third (35.3%) of the twins were monozygotic, the expected proportion of twins who are monozygotic in the United Kingdom general population. The average BMI was 22.3 (SD = 4.2; range = 13.5–59.8), indicating that the sample was relatively lean. A small number reported a vegetarian (*n* = 120; 4.19%), pescatarian (*n* = 77; 2.69%), or vegan (*n* = 20; 0.7%) diet. There were few food allergies. Peanut allergy was the most common (*n* = 54; 1.88%) followed by tree nuts (*n* = 34; 1.19%), wheat/gluten (*n* = 31; 1.08%), and dairy (*n* = 28: 0.98%). A full overview of sample characteristics is shown in **Table 1**.

**TABLE 1:** Demographic characteristics of the study sample

	Sample (*n* = 2865)
Sex, *n* (%)	
Male	1152 (40.21)
Female	1713 (59.79)
Zygosity, *n* (%)	
Monozygotic	1010 (35.25)
Dizygotic	1855 (64.75)
Age, y	19.1 ± 0.3^1^
BMI, kg/m^2^	22.3 ± 4.2
Diet type, *n* (%)	
None	2648 (92.42)
Pescatarian	77 (2.69)
Vegetarian	120 (4.19)
Vegan	20 (0.70)
Food allergy, *n* (%)	
Peanuts	54 (1.88)
Tree nuts	34 (1.19)
Sesame	5 (0.17)
Dairy	28 (0.98)
Shellfish	13 (0.45)
Fish	6 (0.21)
Egg	4 (0.14)
Wheat/gluten	31 (1.08)
Soy	5 (0.17)
Celery	2 (0.07)
Mustard	3 (0.10)
Other^2^	49 (1.71)

1 Mean ± SD (all such values).

2 Includes strawberries, oranges, and apples.

All of the foods on the food preference questionnaire had been tried by >85% of the participants. Mean food item preference scores ranged from 2.28 (SD = 1.39) for cottage cheese (the least-liked food) to 4.70 (SD = 0.64) for chocolate (the most-liked food) (**Table 2**). Pearson’s correlation coefficients indicated that all food-group preference scores were positively associated and that the strongest positive correlation was seen between fruit and vegetable liking (*r* = 0.58, *P* < 0.001). Preference scores for vegetables and snacks had the lowest correlation (*r* = 0.055, *P* = 0.003). The ICCs were higher for monozygotic pairs than for dizygotic pairs for all foods, suggestive of genetic influence on variation in liking for all food groups.

**TABLE 2:** Food item preference scores and ICCs by zygosity^1^

			ICC (95% CI)	
Food item	*n* (%)^2^	Mean preference score^3^ (SD)	Monozygotic	Dizygotic
Vegetables	
Spinach	2686 (94.01)	3.33 (1.42)	0.472 (0.391, 0.545)	0.076 (0.104, 0.251)
Carrots	2851 (99.79)	4.29 (1.01)	0.305 (0.221, 0.384)	0.076 (0.004, 0.147)
Green beans	2816 (98.56)	3.87 (1.24)	0.303 (0.216, 0.385)	0.021 (0.000, 0.093)
Cucumbers	2843 (99.51)	4.04 (1.29)	0.354 (0.271, 0.431)	0.130 (0.059, 0.199)
Celery	2767 (96.95)	2.86 (1.52)	0.464 (0.387, 0.533)	0.227 (0.157, 0.295)
Mushrooms	2826 (98.91)	3.24 (1.67)	0.459 (0.382, 0.529)	0.148 (0.077, 0.217)
Brussels sprouts	2801 (98.04)	2.75 (1.58)	0.471 (0.395, 0.539)	0.145 (0.074, 0.215)
Parsnips	2774 (97.09)	3.35 (1.54)	0.487 (0.411, 0.555)	0.122 (0.050, 0.193)
Peas	2848 (99.69)	4.06 (1.26)	0.273 (0.184, 0.356)	0.062 (0.000, 0.132)
Sweet corn	2842 (99.48)	4.21 (1.19)	0.319 (0.232, 0.401)	0.090 (0.019, 0.159)
Broccoli	2831 (99.09)	4.03 (1.24)	0.371 (0.291, 0.446)	0.080 (0.008, 0.152)
Salad	2848 (99.69)	4.16 (1.08)	0.335 (0.253, 0.412)	0.057 (0.000, 0.128)
Red peppers	2825 (98.88)	4.02 (1.28)	0.423 (0.344, 0.495)	0.114 (0.041, 0.184)
Raw tomatoes	2840 (99.41)	3.29 (1.66)	0.466 (0.392, 0.535)	0.105 (0.035, 0.175)
Avocados	2455 (85.69)	2.73 (1.50)	0.554 (0.479, 0.621)	0.222 (0.146, 0.295)
Potatoes	2860 (99.83)	4.29 (0.95)	0.317 (0.234, 0.395)	0.069 (0.000, 0.138)
Baked beans	2844 (99.30)	3.94 (1.27)	0.317 (0.230, 0.399)	0.085 (0.015, 0.154)
Beetroot	2681 (93.84)	2.70 (1.58)	0.532 (0.459, 0.597)	0.238 (0.166, 0.307)
Fruit	
Oranges	2857 (99.79)	4.30 (1.01)	0.400 (0.322, 0.473)	0.177 (0.109, 0.244)
Grapes	2855 (99.65)	4.62 (0.82)	0.429 (0.350, 0.501)	0.107 (0.038, 0.175)
Apples	2858 (99.83)	4.54 (0.80)	0.553 (0.000, 0.956)	0.000 (0.000, 0.850)
Melon	2839 (99.09)	4.04 (1.29)	0.342 (0.259, 0.421)	0.139 (0.066, 0.210)
Peaches	2795 (97.56)	3.95 (1.26)	0.489 (0.415, 0.556)	0.231 (0.160, 0.298)
Apricots	2736 (95.50)	3.48 (1.37)	0.381 (0.296, 0.460)	0.205 (0.133, 0.275)
Strawberries	2850 (99.58)	4.53 (0.98)	0.460 (0.387, 0.528)	0.082 (0.013, 0.151)
Meat or fish		
Beef	2630 (95.98)	4.36 (0.99)	0.429 (0.342, 0.507)	0.149 (0.070, 0.226)
Beef burgers	2623 (95.64)	4.35 (1.01)	0.350 (0.250, 0.441)	0.123 (0.044, 0.200)
Lamb	2610 (95.01)	3.92 (1.32)	0.508 (0.429, 0.578)	0.244 (0.169, 0.315)
Chicken	2644 (96.61)	4.80 (0.52)	0.192 (0.090, 0.288)	0.090 (0.006, 0.171)
Bacon	2607 (95.11)	4.46 (0.97)	0.361 (0.266, 0.449)	0.051 (0.000, 0.126)
Ham	2608 (95.18)	4.17 (1.05)	0.382 (0.290, 0.465)	0.115 (0.040, 0.189)
Sausages	2632 (96.09)	4.37 (0.96)	0.323 (0.235, 0.405)	0.067 (0.000, 0.146)
White fish	2700 (96.93)	3.97 (1.29)	0.391 (0.301, 0.473)	0.099 (0.018, 0.178)
Canned tuna	2680 (96.19)	3.68 (1.56)	0.553 (0.482, 0.617)	0.226 (0.152, 0.297)
Oily fish	2555 (91.69)	2.74 (1.51)	0.551 (0.477, 0.616)	0.183 (0.106, 0.257)
Smoked salmon	2608 (93.51)	3.31 (1.61)	0.424 (0.337, 0.502)	0.172 (0.095, 0.246)
Hummus	2497 (87.16)	3.15 (1.56)	0.532 (0.451, 0.602)	0.261 (0.185, 0.333)
Dairy	
Eggs	2822 (99.09)	4.13 (1.25)	0.422 (0.342, 0.495)	0.014 (0.000, 0.088)
Soft cheese	2725 (96.54)	3.34 (1.47)	0.484 (0.406, 0.554)	0.206 (0.134, 0.276)
Hard cheese	2803 (99.37)	4.23 (1.12)	0.292 (0.201, 0.378)	0.064 (0.000, 0.140)
Butter	2794 (99.23)	3.96 (1.09)	0.293 (0.205, 0.376)	0.074 (0.000, 0.147)
Cream	2791 (99.02)	3.74 (1.24)	0.266 (0.171, 0.355)	0.013 (0.000, 0.087)
Yogurt	2758 (97.91)	3.63 (1.22)	0.310 (0.221, 0.392)	0.067 (0.000, 0.142)
Cottage cheese	2525 (89.56)	2.28 (1.39)	0.396 (0.305, 0.477)	0.185 (0.108, 0.259)
Butter-like spread	2832 (99.02)	3.78 (1.14)	0.400 (0.320, 0.474)	0.124 (0.051, 0.196)
Mayonnaise	2816 (98.88)	3.56 (1.43)	0.481 (0.406, 0.549)	0.161 (0.091, 0.229)
Custard	2793 (99.48)	3.93 (1.35)	0.494 (0.420, 0.561)	0.203 (0.133, 0.271)
Snacks	
Chips	2861 (99.86)	4.54 (0.76)	0.339 (0.254, 0.418)	0.071 (0.000, 0.143)
Plain biscuits	2854 (99.79)	4.21 (0.91)	0.329 (0.242, 0.410)	0.200 (0.128, 0.270)
Chocolate biscuits	2854 (99.79)	4.56 (0.76)	0.276 (0.185, 0.361)	0.107 (0.033, 0.180)
Cake	2854 (99.79)	4.51 (0.83)	0.179 (0.087, 0.268)	0.140 (0.069, 0.210)
Ice cream	2851 (99.69)	4.52 (0.81)	0.293 (0.207, 0.375)	0.095 (0.024, 0.164)
Chocolate	2855 (99.83)	4.70 (0.64)	0.277 (0.191, 0.358)	0.076 (0.002, 0.150)
Crisps	2855 (99.83)	4.46 (0.83)	0.362 (0.279, 0.439)	0.114 (0.042, 0.185)
Gummy sweets	2833 (99.06)	4.12 (1.11)	0.420 (0.340, 0.493)	0.152 (0.082, 0.221)
Sugared cereal	2851 (99.51)	3.92 (1.12)	0.347 (0.262, 0.426)	0.206 (0.137, 0.273)
Starches				
Bread	2859 (99.83)	4.49 (0.74)	0.193 (0.102, 0.281)	0.070 (0.000, 0.141)
Bran cereal	2790 (97.42)	3.41 (1.24)	0.399 (0.316, 0.476)	0.107 (0.034, 0.177)
Porridge	2811 (98.15)	3.50 (1.36)	0.452 (0.376, 0.521)	0.094 (0.022, 0.165)
Rice	2851 (99.51)	3.95 (1.04)	0.300 (0.215, 0.380)	0.088 (0.016, 0.158)
Wheat cereal	2816 (98.99)	3.98 (1.09)	0.303 (0.217, 0.384)	0.105 (0.033, 0.176)
Rice or corn cereal	2854 (99.62)	4.03 (1.00)	0.292 (0.204, 0.375)	0.090 (0.017, 0.162)

1 ICC, intraclass correlation.

2 “*n*” indicates the number of observations included in the mean food liking score (excluding observations from individuals who reported a restrictive dietary requirement). Percentages reflect the full sample who reported trying the item.

3 Preference scores were rated on a 5-point Likert scale, with a higher score indicating a higher preference for the food item.

Mean food category liking scores are shown in **Table 3**. With a mean preference score of 4.39 (SD = 0.55), snacks were rated as the most popular. In contrast, vegetables were the least-liked group of foods, with a mean preference score of 3.59 (SD = 0.78). In keeping with the patterns of twin correlations for the individual food items, monozygotic pairs were more similar than dizygotic pairs for all 6 food categories, suggestive of genetic influence on food preferences at the group level as well as at the individual food-item level. A broad pattern emerged, which showed that all food category dizygotic within-pair correlations were less than half the monozygotic ICCs, which can indicate the presence of nonadditive genetic factors (D).

**TABLE 3:** Food category preference scores, ICC scores by zygosity, and ACE variable estimates from the model of best fit for the 6 food groups^1^

			ICC (95% CI)	
Food item	*n*	Mean preference score (SD)	Monozygotic	Dizygotic
Vegetables	2865	3.59 (0.78)	0.575 (0.511, 0.632)	0.169 (0.096, 0.237)
Fruit	2862	4.19 (0.80)	0.518 (0.449, 0.581)	0.232 (0.163, 0.298)
Meat or fish	2855	3.89 (0.77)	0.450 (0.374, 0.520)	0.183 (0.110, 0.253)
Dairy	2865	3.62 (0.73)	0.471 (0.399, 0.538)	0.157 (0.086, 0.229)
Snacks	2860	4.39 (0.55)	0.460 (0.385, 0.529)	0.154 (0.082, 0.224)
Starches	2864	3.88 (0.70)	0.362 (0.279, 0.438)	0.084 (0.012, 0.154)

1 Standard ACE model-fitting analyses for continuous data were used. The full ACE model was nested within the saturated model, with subsequent submodels nested within the full ACE model. The selection of the most parsimonious model was indicated by the lowest absolute value of the ACE and the smallest Δχ^2^. Full model-fitting results are summarized in Supplemental Table 2. ACE, Akaike information criterion; ICC, intraclass correlation.

Results from the MLSEM provided more detailed insights into the relative influence of genetic and environmental factors on variations in food preferences. In general, liking for individual food items appeared to be almost entirely explained by genetic influences and the unique environment. For all of the food items, it was possible to drop the shared environmental factor (C), with AE models being preferred in every case. In fact, for almost all foods, the shared environmental effect was estimated to be 0, which indicated no detectable effect of the shared environment on any food preferences in this sample. Heritability estimates for individual food items ranged from 0.18 (95% CI: 0.10, 0.25) for bread to 0.53 (95% CI: 0.46, 0.59) for avocado. The ACE modeling results for each individual food item are presented in full in **Supplemental Table 1**.

**FIGURE 1:**
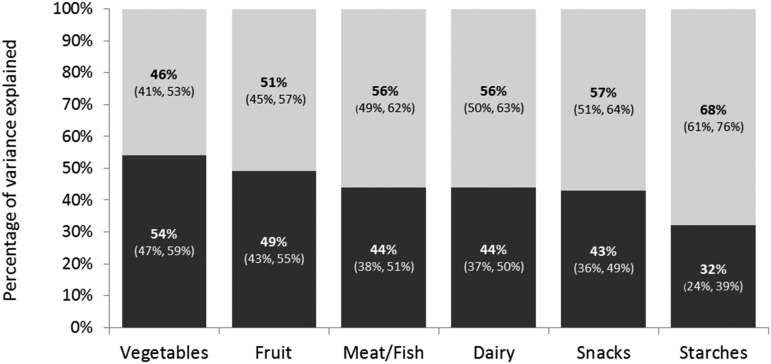
Genetic and environmental influences on food preference categories. Estimates of the percentages in food preference variation explained by genetic factors shown in this figure are based on 2865 participants of the TEDS (Twins Early Development Study) twin cohort. Food preference data were ascertained by self-report with the use of a food preference questionnaire when the participants were 18–19 y old. Genetic influences (“A”), black portion of bars; unique environment influences (“E”), gray portion of bars.

The pattern of the ICCs for monozygotic and dizygotic twins for the different food groups suggested the presence of some nonadditive (D) genetic effects. The use of MLSEM to estimate A, D, and E indicated that variation was explained by additive genetic (A) and nonshared environmental (E) effects, with nonadditive genetic effects (D) being nonsignificant for most food categories. For 5 of 6 food categories, AE models were found to provide the most parsimonious solution. One exception was fruit, with 15% of preference variation explained by dominant genetic effects (D: 0.15; 95% CI: 0.06, 0.24). A full list of estimates and test statistics can be found in **Supplemental Table 2**. Because this study was underpowered to detect small dominant genetic effects (for the majority of the food categories), ACE models were also considered. Estimates for all ACE models with the full model-fitting results are shown in **Supplemental Table 3**. Similar to the ADE solution, the MLSEM results for the food groups that included C instead of D showed no influence of the shared environment on liking for any food group (**Figure 1**). Again, the best-fitting model for each food group was an AE model, which constrained the shared environmental influence (C) to zero.

ACE and ADE models were compared for goodness-of-fit by using the AIC (23). Both ACE and ADE models provided similar fits to the data, apart from vegetable and starch preferences, which favored ADE models (ΔAIC ≥2); ACE and ADE models were of comparable fit for the remaining 4 food categories. Because large sample sizes are needed to detect significant, small, nonadditive genetic effects, we were underpowered to do so. Moderate heritability estimates, obtained from ACE models, were found for liking of most food groups: vegetables (0.54; 95% CI: 0.47, 0.59), fruit (0.49; 95% CI: 0.43, 0.55), meat or fish (0.44; 95% CI: 0.38, 0.51), dairy (0.44; 95% CI: 0.37, 0.50), starches (0.32; 95% CI: 0.24, 0.39), and snacks (0.43; 95% CI: 0.36, 0.49). For each of these food groups, approximately half of the observed variation in preference ratings was accounted for by genetic factors. For all of the food groups the unique environmental effects explained the remaining variance. Significant nonadditive genetic influences (D) were detected for fruit (Supplemental Table 3).

Sensitivity analyses were undertaken to evaluate the impact of self-reported dietary restrictions (e.g., vegetarians or individuals with specific allergies) on ACE estimates for each group. The exclusion of all preference scores for individuals who reported any dietary restrictions (*n* = 358) did not alter the results for any food group. Thus, observations from these individuals were excluded from the analysis only if relevant to the reported diet type or allergy (e.g., vegans and vegetarians were not included in the analyses of preferences for meat or fish). Full details of the sensitivity analysis (*n* = 2309) are shown in **Supplemental Table 4**.

## DISCUSSION

The present study establishes the relative importance of genetic, shared, and unique environmental influences on variations in food preferences in early adulthood. The results show that early shared environmental factors between siblings (e.g., the household or school setting) do not appear to significantly influence food preferences at this older age. Nonetheless, in keeping with previous pediatric and adult studies, we also observed a moderate genetic influence on food preference variations in young adults.

These findings confirm previous research on the etiology of food preferences, which consistently showed a sizeable genetic influence on individual variations in food preferences or intakes (8, 9, 24). Importantly, our results suggest that significant shared environmental influences from childhood are replaced by unique environmental factors by the time individuals enter young adulthood, although longitudinal data from the same sample are needed to test the assumption that shared environmental influences disappear once individuals are able to make autonomous food choices. This is in line with the results of the only other study, to our knowledge, that has investigated genetic and environmental influences on dietary intakes in a sample of young adults (24). In addition, for adolescents, food encounters increasingly occur outside of the family home. The absence of an enduring shared environmental effect has also been documented for other eating behaviors, for example, food intake patterns (14, 25) and general nutrient intake (26–28). Although these findings are broadly consistent with most food preference research undertaken in adults, a small Danish study in adult twins did find significant influences of the shared environment on dietary intake (12). However, these contradictory estimates were derived from food intake data collected by using 1-mo dietary recalls, which is likely to be affected by social desirability bias and have wide 95% CIs.

People intuitively think of cultural influences as playing an important role in shaping food preferences, and many of these cultural influences—both those at the smaller family level as well as at the wider societal level, such as national cuisines—are shared by twin pairs. Finding a substantial influence of the unique environment on food preferences was therefore surprising. However, this observation suggests that twin pairs respond differently to the cultural influences that they are both exposed to. This supports a wealth of research highlighting that children who grow up within the same family experience the same environmental exposures differently (29). Definitive evidence of this developmental change requires a longitudinal study in which the same sample is compared in childhood, adolescence, and adulthood.

The findings of genetic influences on food preferences replicated the results from a previous study in a sample of 3-y-old twins (8). This suggests that food preferences appear early in life and can be reliably measured and that genetic influences on these affinities remain stable over time. The similarities of our food preference heritability estimates compared with this previous study are shown in **Supplemental Figure 1**. Although our results suggest that shared environmental effects detected in early childhood may disappear by late adolescence, the genetic and environmental influences on these traits have not been studied in the same sample at both ages. It is therefore possible that the different estimates of the influence of the shared environment on food preferences could reflect other factors, such as cohort effects.

A substantial heritability of food preferences does not preclude the potential for environmental modification, especially alongside a sizeable environmental influence. Experimental research has shown that repeated exposure to tastes increases flavor acceptance, showing that environmental modification is possible (30–34). To our knowledge, no research has yet investigated the effectiveness, acceptability, or feasibility of a taste modification program in an adult population.

The environment was an important source of influence on food liking in this study (and others), but established drivers of actual food intake, such as cost, availability, and self-regulation, which may also influence food liking, were not accounted for in this study. The ICCs for both the individual food items and the food category scores (Tables 2 and 3) were suggestive of nonadditive genetic effects, because the dizygotic ICCs were less than half the monozygotic ICCs. However, we were unable to detect significant estimates for D (shown in Supplemental Table 3), because we were underpowered to detect these small effects. Nevertheless, given that the D effects were generally small, AE models were a fair representation of the data. Furthermore, liking scores for popular food items such as chocolate were high, with mean scores of <1 SD below the maximum, which may have limited heritability estimates (7). Nonetheless, the food preference measures were able to capture sufficient variance, and the groups with possible ceiling effects did not show significantly different results from the others. Because these findings were established in a predominantly white British and lean twin sample, the extent to which these results may be generalized to the population as a whole may be limited.

However, the large sample size and narrow age range are strengths that allowed reliable estimates for food preferences to be established for a specific developmental phase. A limitation of existing studies in adults is the very wide age range included in each analysis, with studies typically including individuals from early adulthood to older age, making it impossible to ascertain if influences are different for younger and older adults.

It is well established that sweet tastes are universally accepted, and bitter tastes disliked. These dispositions are thought to be the artifacts of an evolutionary adaptive process that facilitated the identification of safe sources of dietary energy and the avoidance of potentially toxic substances (35). However, there is considerable population variation in these preferences, and there is now some molecular genetic evidence to support the heritability estimates observed for variations in some food preferences. Polymorphisms in the Taste 2 receptor gene family (*TAS2R*) genes, a family of 25 bitter-taste receptors, have been associated with variations in sensitivity toward bitter-tasting compounds, such as phenylthiocarbamide (36) and 6-n-propylthiouracil (37, 38). Individuals with a copy of the dominant “taster” PAV haplotype (PAV denotes the encoded amino acid sequence) in the *TAS2R38* gene are most sensitive to 6-n-propylthiouracil (38), and a number of studies have associated lower liking of cruciferous vegetables with this genotype (39, 40). However, more research is needed to identify genetic variants associated with other taste preferences, such as sweet preference. There may also be psychological traits that underlie food preferences. A recent study in 3-y-old British twins established that a substantial proportion of the genetic influence on fruit and vegetable liking could be explained by the genetic influence on food fussiness (41).

Other genetic variants have been identified that are associated with variations in taste perception, but their modes of action are largely unknown. Possible mechanisms include the following: taste receptor density on the tongue (42), reward circuitry (43), and cognitive processes related to self-regulation (44), extraversion (45), food neophobia (46), or anxiety (47), all of which have been associated with food preferences (48).

These results suggest that food preferences are a reasonable target for DNA research. Further research is needed to characterize the biological pathways from genes to behavior. On the other hand, we know a reasonable amount about the environmental shapers of taste preferences.

Food preferences of older adolescents are influenced by both genetic and unique environmental influences. However, our findings of no significant effect of the shared environment on food preferences in adolescence could suggest that children’s early shared family experiences relating to food preferences may not have lasting effects. Overall, our findings indicate that food preferences are approximately equally influenced by genetic, and nonshared, environmental factors. Efforts to improve adolescent nutrition may, for that reason, be best targeted at the wider environment. Strategies might include increasing the availability, lowering the cost, of and promoting “healthier foods” (49). This approach requires stronger government legislation and regulation of the food environment (50).

## Footnotes

6 Abbreviations used: AIC, Akaike information criterion; ICC, intraclass correlation; MLSEM, maximum likelihood structural equation modeling;* TAS2R*, Taste 2 receptor gene family; TEDS, Twin Early Development Study.
